# The Osa-Containing SWI/SNF Chromatin-Remodeling Complex Is Required in the Germline Differentiation Niche for Germline Stem Cell Progeny Differentiation

**DOI:** 10.3390/genes12030363

**Published:** 2021-03-04

**Authors:** Xiaolong Hu, Mengjie Li, Xue Hao, Yi Lu, Lei Zhang, Geng Wu

**Affiliations:** 1 State Key Laboratory of Microbial Metabolism, The Joint International Research Laboratory of Metabolic & Developmental Sciences, School of Life Sciences &Biotechnology, Shanghai Jiao Tong University, Shanghai 200240, China; sw0401@sjtu.edu.cn (X.H.); helios_lmj@163.com (M.L.); 2 State Key Laboratory of Cell Biology, CAS Center for Excellence in Molecular Cell Science, Shanghai Institute of Biochemistry and Cell Biology, Chinese Academy of Sciences, University of Chinese Academy of Sciences, Shanghai 200031, China; haoxue0210@163.com (X.H.); yilu@sibcb.ac.cn (Y.L.); rayzhang@sibcb.ac.cn (L.Z.); 3 School of Life Science and Technology, Shanghai Tech University, Shanghai 201210, China; 4 Bio-Research Innovation Center, Shanghai Institute of Biochemistry and Cell Biology, Chinese Academy of Sciences, Suzhou 215121, China

**Keywords:** germline stem cell, Drosophila ovary, escort cell, differentiation niche, decapentaplegic, engrailed, SWI/SNF, osa

## Abstract

The *Drosophila* ovary is recognized as a powerful model to study stem cell self-renewal and differentiation. Decapentaplegic (Dpp) is secreted from the germline stem cell (GSC) niche to activate Bone Morphogenic Protein (BMP) signaling in GSCs for their self-renewal and is restricted in the differentiation niche for daughter cell differentiation. Here, we report that Switch/sucrose non-fermentable (SWI/SNF) component Osa depletion in escort cells (ECs) results in a blockage of GSC progeny differentiation. Further molecular and genetic analyses suggest that the defective germline differentiation is partially attributed to the elevated *dpp* transcription in ECs. Moreover, ectopic Engrailed (En) expression in *osa*-depleted ECs partially contributes to upregulated *dpp* transcription. Furthermore, we show that Osa regulates germline differentiation in a Brahma (Brm)-associated protein (BAP)-complex-dependent manner. Additionally, the loss of EC long cellular processes upon osa depletion may also partly contribute to the germline differentiation defect. Taken together, these data suggest that the epigenetic factor Osa plays an important role in controlling EC characteristics and germline lineage differentiation.

## 1. Introduction

The self-renewal of adult stem cells and the differentiation of their daughters are critical for homeostasis in many tissues, which are commonly regulated by microenvironments, also known as niches. The niche is composed of several different cell types of somatic lineage, which controls stem cell lineage extrinsically through intercellular signaling pathways [1,2]. It is important to understand how the niche functions to regulate tissue homeostasis.

**Figure 1:**
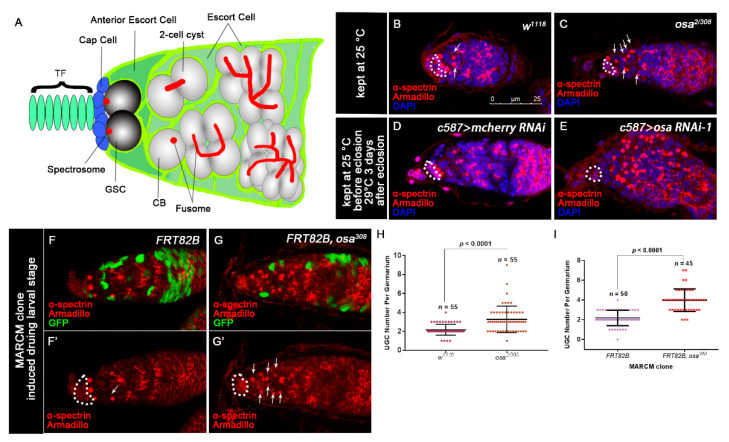
Disruption of Osa results in the accumulation of round fusome-containing germ cells. (**A**) Schematic of a *Drosophila* germarium. (**B**–**E**) Germaria stained for α-spectrin (red), Armadillo (red) and DAPI (blue). CpCs are indicated by white dashed circles. (**B**) The wild-type (WT) (w1118) germarium contains three germline stem cells (GSCs) and two cystoblasts (CBs, indicated by white arrows). (**C**) The *osa^2/308^* germarium displays an expanded number of UGCs outside the GSC niche (indicated by white arrows). The control germarium (**D**) contains a normal number of CBs. The osa KD germarium (**E**) displays an expanded number of UGCs. *c587* > *osa* RNAi-2 gives a similar phenotype and is not shown in the figure. (**F**–**G’**) MARCM clones were stained for α-spectrin (red), Armadillo (red), and GFP (green). Clonal cells are marked by GFP. CpCs are indicated by white dashed circles. (**F**,**F’**) A germarium containing control clones displaying normal number of CBs (indicated by white arrows). (**G**,**G’**) A germarium containing the escort cell (EC) mutant for the *osa^308^* exhibiting an expanded number of UGCs (indicated by white arrows). (**H**) A graph showing the quantification of UGCs for each germarium. (**I**) A graph showing the quantification of UGCs for each germarium. Error bars are presented as the Mean ± SD. Several compressed z-sections are shown in (**B**–**G’**). The scale bar is shown in (**B**).

*Drosophila* germline stem cells (GSCs) and their niches are an attractive system for studying the interactions between stem cells and the niche [3,4]. GSCs reside at the anterior tips of the ovaries in a structure called a germarium. The terminal filament (TF), cap cells (CpCs), and anterior-most escort cells (ECs) form a GSC niche [5,6]. Two to three GSCs are harbored in the niche via physical interactions with CpCs and anterior-most ECs [6,7]. The GSC daughters exit the GSC niche and are then enveloped by the cellular protrusions extended by the ECs, which transport the dividing germline cysts from the anterior portion of the germarium to the posterior [8,9] (Figure 1A). The GSCs are maintained by the Bone Morphogenic Protein (BMP) signaling activity, whose ligand, Decapentaplegic (Dpp), is principally emitted by the CpCs [7,10]. The differentiation factor bam is repressed by the phosphorylated Mothers against dpp (pMad), which is activated by BMP signaling activity [11]. When a GSC daughter cell exits the GSC niche, BMP signaling activity is diminished, and bam transcription is derepressed, which will promote the GSC daughter cell’s differentiation as a cystoblast (CB) [10,11,12,13,14]. The extrinsic cues of germline differentiation mainly come from the differentiation niche constituted by somatic ECs [8,9,15,16,17]. The expression of Thickveins (Tkv) [18] and suppression of Dally [19] in ECs are critical to restrict BMP signaling within a one-cell-diameter range to promote GSC daughter cell differentiation. EC protrusions are also essential for germ cell differentiation [8].

Several epigenetic regulators have been reported to be active in ECs to restrict ectopic BMP signaling in the differentiation niche. Eggless (Egg), a H3K9 methyltransferase in Drosophila [20]; Lysine Specific Demethylase 1 (Lsd1) [21,22]; the piRNA pathway component, Piwi [23,24]; dSet-1 (Set1), a H3K4 trimethylase [25]; Polycomb and Trithorax group genes [26]; and Histone H1 [27] in ECs participate in *dpp* repression and thus secure germline differentiation. The Switch/sucrose non-fermentable (SWI/SNF) complex is a conserved chromatin remolding complex containing two subtypes in *Drosophila*, the Brahma (Brm)-associated protein (BAP) complex and the polybromo-containing BAP (PBAP) complex [28]. Brm, Moira (Mor), and Snf5-related 1 (Snr1) are the common components of both subtypes [29,30]. Brm is the catalytic subunit containing DNA-stimulated ATPase, while Moira and Snr1 are required for stable complex assembly [31,32,33,34]. Osa is specific to the BAP complex and is required for the recruitment of the complex to specific loci [35]. Brahma-associated protein 170kD (Bap170) and Polybromo are the signature subunits of the PBAP complex [35,36]. Osa has been reported to be an important epigenetic regulator for *Drosophila* development. In the *Drosophila* adult midgut, Osa activates the transcription of *Delta* (*Dl*) in intestinal stem cells (ISCs), which in turn stimulates the Notch signaling activity in enteroblast and promotes an enterocyte fate commitment [37]. In the Drosophila neural stem cell lineage, Osa limits intermediate neural progenitors (INPs), thus regaining neuroblast properties, and prevents brain tumor formation [38]. In eye imaginal discs, Osa interacts genetically and biochemically with Cyclin E to promote cell cycle progression [39]. In the wing disc, Osa is needed for vein patterning through regulating gene expression in response to Epidermal Growth Factor Receptor (EGFR) signaling and cooperation with Groucho (Gro) and Histone deacetylase 1 (Rpd3) to repress Wingless target genes [40,41]. In mammals, the mammalian homolog of Osa, ARID1A, contributes to liver regeneration in hepatocytes and has also been identified as a tumor suppressor in human cancers [42,43,44,45].

In this paper, we report evidence that Osa participates in GSC progeny differentiation in a BMP-signaling-dependent manner. Osa participates in the repression of dpp transcription in ECs, which facilitates BMP signaling activity outside the GSC niche. We also show that Osa represses Engrailed (En) expression in ECs. The ectopic En in *osa*-depleted ECs also partially contributes to activating the transcription of *dpp*. Furthermore, Osa regulates germline differentiation in a BAP-complex-dependent manner. Meanwhile, Osa controls EC protrusions. In addition, the adult EC knockdown of *osa* was observed to induce GSC loss. Taken together, our findings support that the epigenetic factor Osa plays an important role in controlling EC characteristics and germline lineage differentiation.

## 2. Materials and Methods

### 2.1. Fly Strains and Genetics

Flies were cultured at 25 °C on standard cornmeal media supplied with live yeast to the food surface unless otherwise stated.

To maximize the RNAi-mediated knockdown effect, newly enclosed flies were cultured at 29 °C for 3 days before dissection.

For pupal stage-specific expression of RNAi, *c587* was used in combination with *tubP-GAL80*^ts^ (*c587ts* for short), crosses were set up, and the progenies were kept at 18 °C until the early pupal stage before being transferred to 29 °C. The newly born females (<1 day old) or 3-day-old females were dissected for analysis.

For the adult-specific expression of RNAi, *c587ts* was used, crosses were set up, and the progenies were kept at 18 °C until eclosion, before being transferred to 29 °C for another 14 days prior to dissection.

Strains used in this study were as follows: *w^1118^* was used as a control; *osa^308^*(BDSC 5949), *osa^2^*(BDSC 3616), *nos-gal4*(gift from Yu Cai), *c587-gal4* (gift from Yu Cai), *dad-lacZ* (gift from Yu Cai), *bamP-GFP* (gift from Dennis M McKearin), *dpp^hr4^* (gift from Ting Xie), *dpp^e90^* (gift from Ting Xie), *dpp2.0-lacZ* (gift from Yu Cai), *en^4^* (BDSC 1817), *en^7^* (BDSC 1820), *dpp* RNAi-1 (BDSC 31531), *dpp* RNAi-2 (BDSC 31530), *en* RNAi-1 (VDRC 105678), *en* RNAi-2 (BDSC 33715), *mcherry* RNAi (BDSC 35785), *hh-lacZ* (BDSC 5330), *UAS-mCD8-GFP* (BDSC 5137), *osa* RNAi-1 (BDSC 31266), *osa* RNAi-2 (BDSC 35447), *brm* RNAi-1 (BDSC 34520), *brm* RNAi-2(BDSC 31712), *mor* RNAi-1 (BDSC 34919), *mor* RNAi-2 (VDRC 6969), *snr1* RNAi (BDSC 32372), *bap170* RNAi (BDSC 26308), *bap180* RNAi-1 (BDSC 32840), *FRT82B* (BDSC 8216), and *tubP- GAL*80^ts^ (BDSC 7017).

### 2.2. Clone Generation

MARCM clones of the osa mutant and control were generated by crossing FRT82B, osa^308^, or FRT82B with hs-Flp/FM7; UAS-GFPnls, tubP-GAL4/Cyo, y^+^; and tubP-GAL80, FRT82B.

The resulting larvae were heat shocked at 37 °C with three 60 min pulses at 5 h intervals each day from the mid-third larval instar (ML3, 96 h after egg laying (AEL)) to early pupal stage (EP, 144 h AEL). After heat shock, the flies were kept at 29 °C until dissection. Newly born (<1 day old) females were dissected and stained with the appropriate antibodies.

### 2.3. Pupae Staging

Staging of the pupae was performed as reported [46,47]. In short, synchronized eggs were collected in a fresh vial within 2 h. After the parents were removed, the offspring were cultured with optimal nutrition and uncrowded conditions. For flies carrying *tub-Gal80^ts^*, vials were kept at 18 °C until the temperature shift. Under these conditions, the EP was 12 days AEL.

### 2.4. Antibodies and Immunofluorescence

Staining of the ovaries was carried out as described previously [48]. In brief, females were dissected in PBS and fixed in 4% paraformaldehyde (Sigma, Darmstadt, Germany) in PBS for 30 min, rinsed with 0.3% PBST (PBS containing 0.3% Triton X-100 (Bio-Rad, Hercules, CA, USA)) three times, permeated with 1.0% PBST for 1 h, blocked with 10% goat serum (Life Technology, New York, NY, USA) in 0.3% PBST for 2 h, and stained overnight at 4 °C with primary antibodies. The ovaries were then washed three times in 0.3% PBST and incubated with secondary antibodies for 2 h at room temperature and stained with DAPI for 10 min. Finally, the ovaries were rinsed four times with 0.3% PBST and mounted.

The following primary antibodies were used in this work: Mouse anti-α-spectrin (1:20, DSHB 3A9), mouse anti-Armadillo (1:20, DSHB N2 7A1), rabbit anti-β-gal (1:1000, Cappel, Cochranville, PA, USA), rabbit anti-pMad (1:500, gift from ED. Laufer), rabbit anti-GFP (1:1000, Life Technology, New York, NY, USA), mouse anti-Engrailed (1:10, DSHB 4D9), rabbit anti-Engrailed (1:200, Santa Cruz, SCZ, USA), mouse anti-Osa (1:5, DSHB Osa). Secondary antibodies conjugated with Alexa Fluor 488 or 546 (Life Technology, New York, NY, USA) were used at 1:1000 dilutions. DAPI (Life Technology, New York, NY, USA,) was used to visualize the nuclei. Confocal images were captured on a Leica TCS SP8 WLL laser confocal microscope and obtained using the Leica AF Lite system (Leica, Weztlar, Germany). Images were processed in Adobe Photoshop CS6 (Version: 13.0 x 32).

### 2.5. Measurement of Fluorescence Intensity

To compare the fluorescence intensity between the gene knockout ECs and the control, all images were measured under the same parameters at the same time using the Leica SP8 quantification software (Version: LAS-AF-Lite_2.6.0). The selected regions within the ECs were randomly picked, and the Osa-staining mean fluorescence intensity was normalized to the fluorescence intensity of the adjacent germline cells, calculated, and compared. The *hh-lacZ* mean fluorescence intensity was normalized to the background, calculated, and compared.

### 2.6. EC Purification, RNA Isolation, and Quantitive PCR

GFP-positive ECs were isolated from the ovaries of *c587-gal4/+*; *uas-mCD8-GFP/+*; *osa RNAi/+* and *c587-gal4/+*; *uas-mCD8-GFP/+*; and *mcherry RNAi/+* virgins using FACS (BD FACSAria III, New Jersey, NJ, USA) according to the procedure published previously [19,49]. Total RNA was isolated with Trizol according to the manufacturer’s manual. cDNA was synthesized using a TOYOBO FSQ-301 kit (Osaka City, Japan), and qPCR was performed using SYBR Green (TOYOBO QPK-201, Osaka City, Japan) on a LightCycler 96 (Roche, Basel, Switzerland). The 2^−ΔΔ*C*t^ method was used to compare the gene expression levels. *dpp* RNA levels were normalized to *rp49*. Each reaction was performed in triplicate. Three biological replicates were performed.

### 2.7. Statistical Analysis

**Figure 2:**
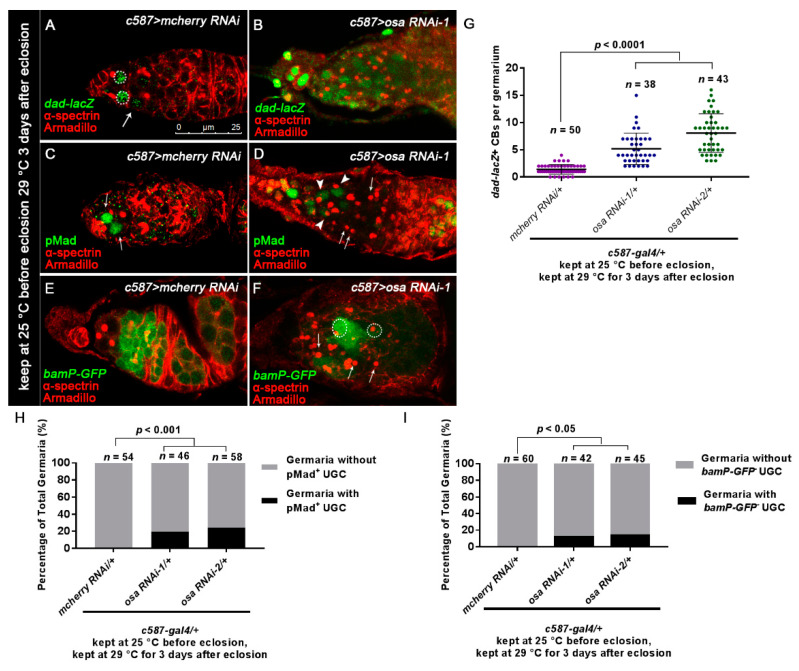
Loss of osa in ECs results in expanded BMP signaling. (**A**,**B**) Germaria stained for α-spectrin (red), Armadillo (red), and β-gal (green). BMP signal activity was monitored by *dad-lacZ* (detected by β-gal). The control germarium (**A**) exhibited three GSCs (indicated by white dashed circles) and one CB (indicated by white arrows) positive for *dad-lacZ*. The *osa RNAi-1* germarium (**B**) exhibited *dad-lacZ*-positive UGCs. (**C**,**D**) Germaria were stained for α-spectrin (red), Armadillo (red), and pMad (green). The control germarium (**C**) exhibited strong pMad staining in GSCs (indicated by white arrows). In the *osa RNAi-1* germarium (**D**), some of the accumulated UGCs exhibited pMad staining (indicated by white arrowheads), while others were negative for pMad (indicated by white arrows). (**E**,**F**) Germaria stained for α-spectrin (red), Armadillo (red), and GFP (green). *bam* transcription was monitored by *bamP-GFP* (detected by GFP). In the control germarium (**E**), *bamP-GFP* was negative in GSCs and upregulated in CB and the dividing cysts. In *osa RNAi-1* germarium (**F**), there is a mixture of *bamP-GFP*-positive (indicated by white dashed circles) and *bamP-GFP*-negative (indicated by white arrows) UGCs. *c587* > *osa RNAi-2* has a similar phenotype (not shown). Several compressed *z*-sections are shown in (**A**–**F**). (**G**) Graph showing the quantification of *dad-lacZ*-positive UGCs for each germarium. (**H**) Graph showing the percentage quantification of germaria with pMad-positive UGCs. (**I**) Graph showing the percentage quantification of germaria with *bamP-GFP*-negative UGCs. The scale bar is shown in (**A**).

**Figure 3:**
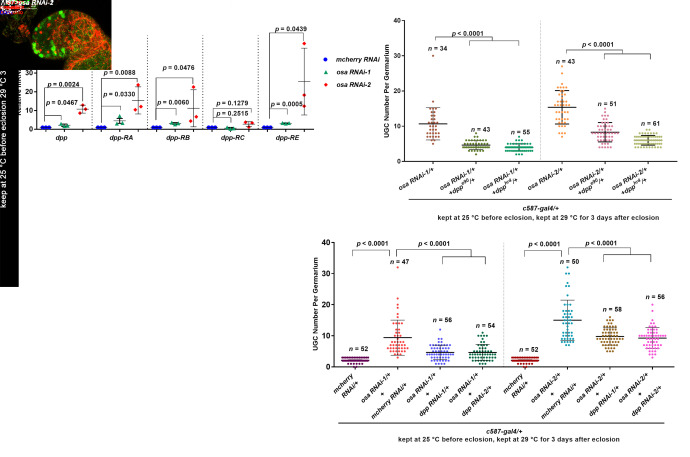
Osa limits dpp transcription in ECs for germline differentiation control. (**A**) Quantitative PCR results for the purified ECs showing that mRNA levels for the annotated dpp transcripts were upregulated in osa KD ECs. (**B**,**C**,**D**) Germaria stained for α-spectrin (red), Armadillo (red), and β-gal (green). dpp transcription was monitored by dpp2.0-lacZ (detected by β-gal). CpCs are indicated by white dashed circles. In the control germarium (**B**), dpp2.0-lacZ was expressed in CpCs. In the osa RNAi-1 (**C**) and osa RNAi-2 (**D**) germaria, dpp2.0-lacZ was also expressed in ECs (indicated by white arrows). (**E**–**K**) Germaria stained for α-spectrin (red), Armadillo (red), and DAPI (blue). (**H**) The control germaria exhibiting a normal number of CBs. The osa RNAi-1 (**E**) and osa RNAi-1+ mcherry RNAi (**I**) germaria exhibiting UGC accumulation. The introduction of one copy of the dpp allele (**F**,**G**) into the background of c587 > osa RNAi-1 or dpp RNAi (**J**,**K**) into the background of c587>osa RNAi-1 relieved the UGC accumulation phenotype. c587>osa RNAi-2 provided similar phenotypes (not shown). (**L,M**) Graphs showing quantification of the UGC number per germarium. Error bars are presented as the Mean ± SD. Several compressed z-sections are shown (**B**–**K**). The scale bar is shown in (**B**).

**Figure 4:**
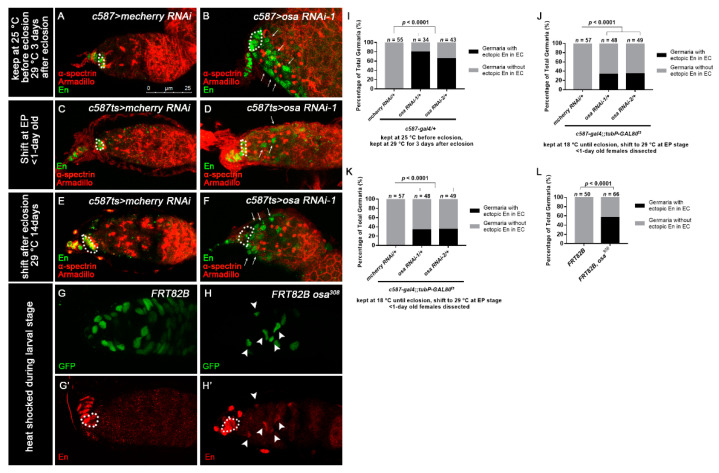
Osa represses *engrailed* expression in ECs. (**A**–**F**) Germaria stained for α-spectrin (red), Armadillo (red), and En (green). (**A**,**B**) Flies were initially kept at 25 °C and then shifted to 29 °C after eclosion. (**C**–**F**) *c587-gal4;;tubP-GAL80^ts^* was used for *osa* KD. (**C**,**D**) Flies were initially kept at 18 °C and then shifted to 29 °C at the EP stage. The newly born females were dissected. (**E**,**F**) Flies were initially kept at 18 °C until eclosion and then kept at 29 °C for another 14 days before dissection. The control germaria (**A**,**C**,**E**) exhibited specific En staining in TF and CpCs. The *osa* *RNAi-1* germaria (**B**,**D**,**F**) exhibited ectopic En staining in the ECs (indicated by white arrows) in addition to CpCs. *c587* > *osa RNAi-2* gave a similar phenotype (not shown). (**G**–**H’**) MARCM clones stained for En (red). Clonal cells were marked by GFP (green). CpCs here are indicated by white dashed circles. The mosaic germarium with control clones (**G**,**G’**) exhibited specific En staining in TF and CpCs. The mosaic germarium with *osa^308^* mutant ECs (**H**,**H’**) exhibited ectopic En staining in clonal ECs (indicated by white arrow heads) in addition to CpCs. Several compressed *z*-sections are shown in (**A**–**H’**). (**I**–**L**) Graph showing the percentage quantification of germaria with En ectopic expression. The scale bar is shown in (**A**).

**Figure 5:**
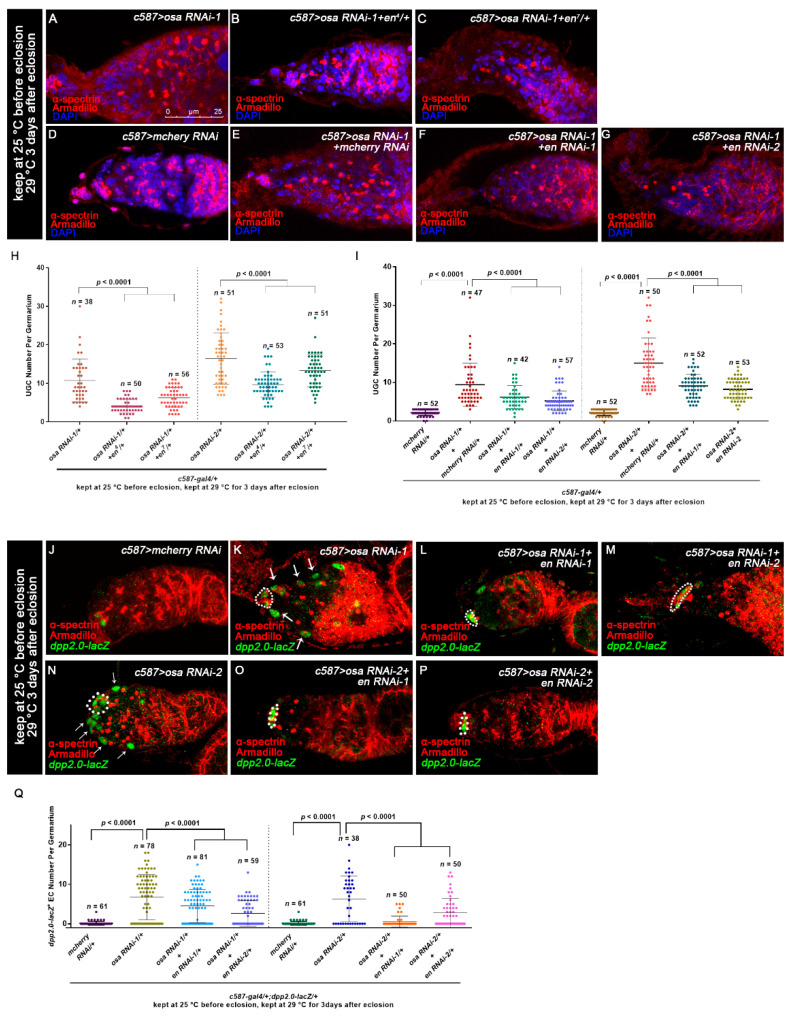
Osa regulates *dpp* partially through Engrailed. (**A**–**G**) Germaria stained for α-spectrin (red), Armadillo (red), and DAPI (blue). The osa RNAi-1 (**A**) and osa RNAi-1+mcherry RNAi (**E**) germaria exhibited UGC accumulation. (**D**) The mcherry RNAi germaria exhibited a normal number of CBs. Introduction of one copy of an en allele (**B**,**C**) or en RNAi (**F**,**G**) significantly relieved the germ cell differentiation defect. (**H**,**I**) Graphs show the quantification of UGC number per germarium. Error bars are presented as the Mean ± SD. (**J**–**P**) Germaria stained for α-spectrin (red), Armadillo (red), and β-gal (green). dpp transcription was monitored by dpp2.0-lacZ (detected by β-gal). CpCs are indicated by white dashed circles. In the control germarium (**J**), *dpp2.0-lacZ* was expressed in CpCs. In osa RNAi-1 (**K**) and osa RNAi-2 (**N**) germaria, dpp2.0-lacZ was ectopically expressed in ECs (indicated by white arrows) in addition to CpCs. The introduction of en RNAi-1 (**L**,**O**) or en RNAi-2 (**M**,**P**) reduced the ectopic dpp2.0-lacZ in ECs. (**Q**) A graph showing the quantification of dpp2.0-lacZ-positive ECs for each germarium. Error bars are presented as the Mean ± SD. Several compressed z-sections are shown in (**A**–**G**,**J**–**P**). The scale bar is shown in (**A**).

**Figure 6:**
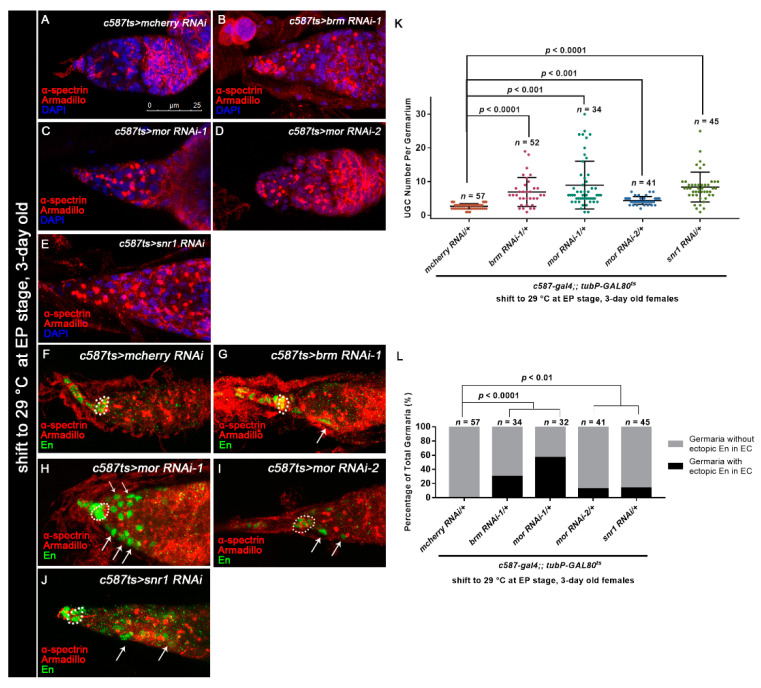
Osa functions in a BAP complex-dependent manner. (**A**–**E**) Germaria stained for α-spectrin (red), Armadillo (red), and DAPI (blue). *c587-gal4;;tubP-GAL80^ts^* was used for BAP complex component KD. The germarium from the control group (**A**) exhibited a normal number of CBs. The *c587ts* > *brm RNAi-1* (**B**), *c587ts* > *mor RNAi* (**C**,**D**), and *c587ts* > *snr1 RNAi* (**E**) germaria exhibited UGC accumulation. (**K**) Graph showing the quantification of UGCs in each germarium. Error bars are presented as the Mean ± SD. (**F**–**J**) *c587-gal4;;tubP-GAL80^ts^* was used for BAP complex component KD. Germaria were stained for α-spectrin (red), Armadillo (red), and En (green). CpCs are indicated by white dashed circles. Germaria from the control group (**F**) exhibited exclusive expression of En in TF and CpCs. Germaria from *c587ts* > *brm RNAi-1* (**G**), *c587ts* > *mor RNAi* (**H**,**I**), and *c587ts* > *snr1 RNAi* (**J**) exhibited ectopic En expression in ECs (indicated by white arrows) in addition to CpCs. (**L**) Graph showing the percentage quantification of germaria with En ectopic expression. Several compressed *z*-sections are shown in (**A**–**J**). The scale bar is shown in (**A**).

**Figure 7:**
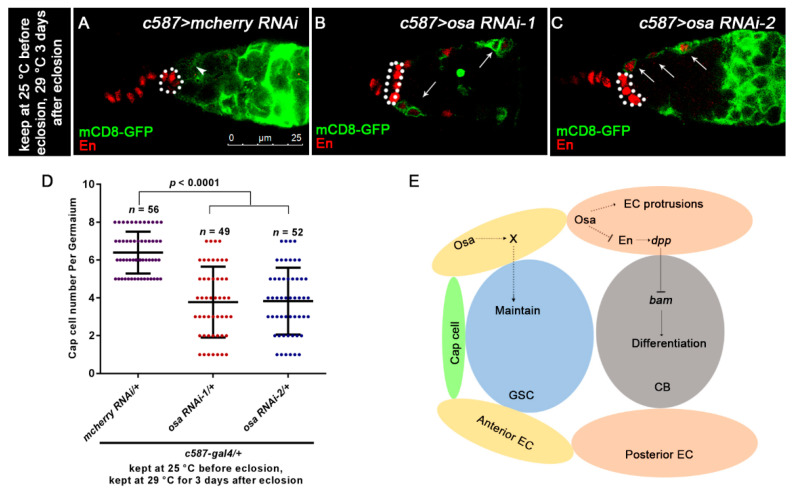
Osa regulates EC characteristics. (**A**,**C**) Germaria are stained for En (red) and GFP (green). EC membranes are marked by mCD8-GFP (detected by GFP). CpCs are indicated by white dashed circles. In control germarium (**A**), EC protrusions extend between cysts (indicated by white arrowhead). Additionally, *en* is exclusively expressed in TF and CpCs. In *osa* *RNAi-1, osa* *RNAi-2* germarium (**B**,**C**), ECs lose their cellular processes (indicated by white arrows). Additionally, *en* is ectopically expressed in ECs, which is also marked by GFP (indicated by white arrows). (**D**) Graph shows the quantification of CpC per germarium. Error bars are presented as Mean ± SD. (**E**) Model illustrates Osa-mediated regulation of GSC maintenance and germ cell differentiation in ECs in germaria. Scale bar is shown in (**A**).

All statistical data were recorded in Excel (Microsoft, Redmond, WA, USA) and graphed in Prism 7.0 (GraphPad Software, Version: 7.00). Figure 1H,I: Student’s *t* test was used to compare the significant difference between two group. Figure 2H,I: chi-squared test was used when analyzing the categorical variable data. Figure 2G: one-way ANOVA test was applied when analyzing more than two groups. Figure 3A: paired-sample *t*-tests were used to compare the qRT-PCR results. Figure 3L,M: one-way ANOVA test was applied. Figure 4I–L: chi-squared test was used. Figure 5H,I,Q: one-way ANOVA test was applied. Figure 6K: one-way ANOVA test was applied. Figure 6L: chi-squared test was used. Figure 7D: one-way ANOVA test was applied. Figure S1D: one-way ANOVA test was applied. Figure S2E,I,J: one-way ANOVA test was applied. Figure S3C,D: Student’s *t* test was used. Figure S3E: one-way ANOVA test was applied. Figure S4C–E: one-way ANOVA test was applied. A value of *p* < 0.05 was considered statistically significant.

## 3. Results

### 3.1. Osa Is Required in Escort Cells for GSC Progeny Differentiation

Since Osa is an important epigenetic regulator for *Drosophila* development, we sought to identify whether Osa regulates the development of the germline. To evaluate the germline differentiation status in both wild-type (WT) and *osa* mutants (*osa^2/308^*), we stained the ovaries with α-spectrin and Armadillo to mark the spectrosome/fusome and the CpCs, respectively. The GSCs were identified by the spectrosomes, which directly contact the CpCs. Additionally, CBs were identified by round fusomes, which do not reside immediately adjacent to the CpCs [50] (Figure 1A). The control germaria typically contain 0–4 CBs outside the GSC niche (Figure 1B,H, white arrows). In contrast, the *osa^2/308^* germaria contained significantly rounder fusome-containing germ cells than the control germaria (Figure 1C,H, white arrows). For quantification, the round fusome-containing germ cells outside the GSC niche were quantified as undifferentiated germ cells (UGCs). Altogether, these results indicate that decreased *osa* expression impairs differentiation of the germ cells.

GSC progeny differentiation is also controlled extrinsically by the differentiation niche formed by ECs. Thus, we sought to identify if Osa is required non-cell autonomously within ECs for GSC progeny differentiation. We first used *c587-gal4*, which is expressed in most somatic lineages in developing gonads but becomes largely restricted in ECs and early follicle cell progenitors in adulthood [51,52], to drive the expression of *osa* RNAi in ECs. Two individual RNAi lines (*osa* RNAi-1 and *osa* RNAi-2) were used to knockdown (KD) *osa* expression. To maximize KD efficiency moderately, the flies were initially kept at 25 °C until eclosion and then transferred to 29 °C for 3 days prior to dissection. First, the knockdown efficiency of two RNAi lines was confirmed by immunostaining. In the control germaria, Osa was ubiquitously expressed in both germline and somatic lineages, as detected by the Osa antibody (Figure S1A,A’, white arrows). In *osa* RNAi germaria, *osa* KD led to faint Osa staining in ECs (Figure S1B–D, white arrows). In addition, *osa* KD also induced UGC accumulation in the germaria (Figure 1D,E). These results indicate that knockdown *osa* in ECs may block germ cell differentiation.

It was previously reported that at two hours after puparium formation (APF), germaria were formed, and ECs could be identified by Tj and LamC antibody staining; at 48 h APF, germaria were completely formed [53]. Since *c587-gal4* is known to be expressed in both somatic precursors and adult ECs [54], to avoid the influence of *osa* knockdown in somatic precursors before EC formation, we combined *c587-gal4* with a temperature-sensitive mutant *gal80* (*tubP- GAL*80^ts^) (referred to *c587ts* for short) to temporally control *osa* KD. GAL80^ts^ is active at 18 °C and represses the activity of GAL4 but is inactive at 29 °C; thus, GAL4 could initiate the expression of RNAi [55]. First, we carried out the pupae-stage-specific KD of *osa*. To bypass the potential requirement of Osa before germarium formation, flies were initially kept at 18 °C and transferred to 29 °C during the early pupal stage after EC formation. The newly born females (<1 day old) were checked. Compared with the control germaria, the expression of *osa* RNAi led to a dramatic expansion of UGCs (Figure S2A,B,E). For the adult-specific KD, flies were initially kept at 18 °C until eclosion and then shifted to 29 °C for another 14 days before dissection. Compared with the control germaria, *osa* KD germaria were also filled with UGCs (Figure S2C–E). To rule out the leaky expression of *osa* RNAi before eclosion, the newly eclosed females were also checked, and their germaria appeared normal (Figure S2F–I). These findings suggest that Osa is required in ECs for germline differentiation control after EC formation. Interestingly, in addition to UGC accumulation upon the adult-specific KD of *osa*, these germaria also exhibited a significant GSC loss phenotype (Figure S2J). Since a recent study suggested that the anterior ECs are essential for GSC maintenance [6], we inferred that *osa* may be required in anterior ECs for GSC maintenance in adulthood.

To confirm the phenotypes of *osa* transheterozygotes and *c587-gal4-*driven *osa* RNAi, we performed a clonal analysis by inducing positively marked *osa^308^* clones using the MARCM system [56] and then evaluated the UGC quantity in the mosaic germaria. Both the control and mutant clones were induced in parallel. The mosaic germaria with control clones (*FRT82B*) exhibited a normal number of CBs (Figure 1F,F’,I). However, the mosaic germaria with *osa^308^* clonal ECs presented accumulated UGCs (Figure 1G,G’,I, white arrows). It was reported that *brm*’s loss of function in the germline does not disrupt germline differentiation [57]. Moreover, no UGC accumulation phenotype was found when we used *nos-gal4* to knock down *osa* in the germline (unpublished data). These results indicate that Osa is required in ECs for germ cell differentiation control.

### 3.2. Osa Is Required for the Restriction of BMP Signaling Outside the GSC Niche

Previous studies have reported that the inactivation of BMP signaling is required for the differentiation of GSC daughters. For this purpose, we checked *dad-lacZ*, an enhancer trap line for the target gene of the BMP pathway [58]. Normally, *dad-lacZ* is confined to GSCs and CBs [59] (Figure 2A, white dashed circles and white arrows). By contrast, the number of *dad-lacZ*-positive UGCs was expanded in *c587-gal4-*driven *osa* KD germaria, although the intensity of β-gal staining was not as high as that in the control germaria (Figure 2B,G).

Activation of BMP signaling in GSCs resulted in the phosphorylation of Mad (pMad). Then, pMad translocated into the GSC nucleus and repressed the differentiation factor *bam* [11,14,60]. pMad staining is normally restricted in GSCs (Figure 2C, white arrows). In the germaria of *osa* KD, pMad staining was strong in the GSCs. A part of UGCs in a subset of germaria expressed pMad at a low level (Figure 2D, white arrowheads; Figure 2H, osa RNAi-1 germaria(9/46), osa RNAi-2 germaria(14/58)).

Next, we monitored the *bam* transcription activity with *bamP-GFP* (a GFP reporter of *bam* transcription) [60]. *bamP-GFP* expression is normally absent in GSCs; then, it becomes detectable in CB and reaches its highest levels in dividing cysts (Figure 2E). However, in a subset of germaria with *osa* KD, several UGCs were *bamP-GFP*-negative outside the GSC niche (Figure 2F, white arrows), while others exhibited upregulated *bamP-GFP* expression (Figure 2F, white dashed circles). 11.9%(5/42) of *osa* RNAi-1 germaria and 13.8% (9/65) of osa RNAi-2 germaria contained bamP-GFP negative UGCs (Figure I). These results indicate that the accumulated UGCs represent a mixture of GSC-like and/or CB-like stages. Collectively, *osa*-depletion in ECs induces ectopic BMP signaling activity outside the GSC niche.

### 3.3. Osa Restricts Dpp Transcription in Escort Cells

There is growing evidence that Dpp is regulated precisely in ECs to facilitate homeostasis of the germline [18,22,26,61]. Moreover, the upregulation of *dpp* transcription in ECs is responsible for the enlargement of BMP activity outside the GSC niche. To determine if Osa is required in ECs to repress *dpp* transcription, we performed qRT-PCR to examine the transcription levels of *dpp* in the FACS-sorted control and *osa* KD ECs. The primer sequences are provided in Table 1. Since there are four annotated *dpp* isoforms in *Drosophila*, first, we detected the *dpp* mRNA level using primer pairs recognizing all four isoforms. Then, *dpp* was increased in the EC samples with *osa* KD than that of control ones. To determine if the elevation is isoform-specific, we next examined *dpp* expression using isoform-specific primer pairs. We found significant upregulation of *dpp-RA*, *dpp-RB*, and *dpp-RE* in *osa* KD ECs (Figure 3A). These results indicate that Osa is required in ECs to repress *dpp* transcription.

To visualize *dpp* expression in the germaria, we introduced *dpp2.0-lacZ* [62], which is used to monitor *dpp* transcription, into the context of the *c587-gal4-*driven *osa* KD. As previously reported, *dpp2.0-lacZ* is highly expressed in CpCs (Figure 3B, white dashed circle) and sporadically expressed in ECs in control germaria [62]. However, *osa* KD induced the ectopic expression of *dpp2.0-lacZ* in not only the anterior-most ECs but also in the posterior ones (Figure 3C,D, white arrows). Collectively, these results indicate that *dpp* transcription is upregulated in *osa*-deficient ECs.

To further demonstrate the link between elevated *dpp* in ECs and UGC formation, we performed a genetic analysis by reducing *dpp* in the background of *c587-gal4-*driven *osa* KD and assessed the UGC number. We found that either of the two *dpp* alleles, *dpp^e90^* or *dpp^hr4^*, could partially rescue the UGC accumulation phenotype induced by *osa* KD (Figure 3E–G,L). Consistently, the double knockdown of *osa dpp* also partially reduced the UGC number in *osa* KD germaria (Figure 3H–K,M). In summary, these results show that Osa regulates GSC progeny differentiation partly by modulating *dpp* in ECs.

### 3.4. Osa Limits Engrailed Expression in Escort Cells

Next, we sought to investigate how Osa limits *dpp* expression outside the GSC niche. It was reported that Osa may contribute to restricting *engrailed* (*en*) expression in embryo [63], indicating that Osa is a repressor of *en*. Meanwhile, ectopic expression of *en* in ECs leads to ectopic BMP signaling, resulting in an expanded GSC-like cell tumor phenotype [22], and En regulates *dpp2.0-lacZ* reporter activity in CpCs [62]. Thus, we tested whether *en* is upregulated in ECs upon *c587-gal4-*driven *osa* KD. For this purpose, we carried out immunostaining of En in the germaria of the control and *osa* KD females. Flies were initially kept at 25 °C until eclosion and then transferred to 29 °C for another 3 days prior to dissection. The control germaria exhibited the specific expression of En in TF and CpCs (Figure 4A, white dashed circle). Interestingly, some *c587-gal4-*driven *osa* KD germaria exhibited ectopic En expression in ECs (*osa RNAi-1*: 27/34 of total germaria, *osa RNAi-2*: 28/43 of total germaria, Figure 4B, white arrows; Figure 4I).

We subsequently investigated temporally controlled manipulations of *osa*. For the pupal-stage-specific expression of RNAi, flies were initially kept at 18 °C until the early pupal stage and then transferred to 29 °C for RNAi expression. The newly born females (<1 day old) were checked. For the adult-specific expression of RNAi, flies were raised at 18 °C and then shifted to 29 °C after eclosion. The flies were aged at 29 °C for 14 days prior to dissection. In the control germaria, En was restricted to the TF and CpC (Figure 4C,E, white dashed circles). Surprisingly, the germaria of both pupal-specific and adult-specific *osa* depletion exhibited ectopic *en* expression in the ECs (pupae-specific *osa* KD: *osa RNAi-1*: 16/48 of total germaria, *osa RNAi-2*: 17/49 of total germaria; adult-specific *osa* KD: *osa RNAi-1*: 51/51 of total germaria, *osa RNAi-2*: 58/59 of total germaria; Figure 4D,F, white arrows; Figure 4J,K). Then, we further examined the *en* expression patterns in the *osa* mosaic germaria. *en* was also ectopically expressed in *osa* mutant clonal ECs (37/66 of total clonal germaria, Figure 4G–H’, white arrowheads; Figure 4L), which confirmed the results of the RNAi KD. These results suggest that Osa may repress *en* expression in ECs after EC formation in both the pupal stage and adulthood.

To test if the upregulated *en* in ECs is essential for the UGC formation induced by *osa* KD, we reduced *en* expression by introducing one copy of the *en* allele into the background of the *osa* RNAi. Indeed, both *en^4^* and *en^7^* could partially suppress the UGC accumulation induced by *osa* KD (Figure 5A–C,H). A similar effect was also found when *en* RNAi was co-expressed with *osa* RNAi (Figure 5D–G,I). In summary, the ectopic expression of *en* in ECs is partially attributed to the UGC accumulation in *c587*-driven *osa* KD germaria.

To further determine if the ectopic expression of *en* in ECs is responsible for the ectopic *dpp2.0-lacZ* activity caused by *osa* KD in ECs, we co-expressed *en* RNAi and *osa* RNAi with *c587-gal4* and then quantified *dpp2.0-lacZ* activity. Interestingly, two individual *en* RNAi lines could partially suppress the ectopic β-gal staining in ECs in comparison with *osa* KD alone (Figure 5J–Q). These results indicate that ectopic expression of *en* is at least partially responsible for the increased *dpp* transcription caused by *osa* KD in ECs.

### 3.5. Osa Regulates Germline Differentiation through the BAP Complex

Osa is a component of the BAP complex, which also includes Brm, Snr1, and Mor. Therefore, we wondered if Osa regulates GSC progeny differentiation in a BAP-complex-dependent manner. To assess this possibility, we tested *c587-gal4-*driven RNAi against *brm*, *snr1,* or *mor*. Since overexpression of these RNAi lines (*brm* RNAi-1, *snr1* RNAi-1, *mor* RNAi-1) with *c587-gal4* caused pupal lethality or the emergence of abnormal ovaries at 25 °C, we used *c587ts* to bypass the potential developmental effects. Flies were initially kept at 18 °C and then transferred to 29 °C at the early pupal stage until dissection. Germaria from 3-day-old females exhibited significant expansion of UGCs in the germaria, which was similar to the effects in *osa* KD (Figure 6A–E, Figure S2A,B,E). The *c587-gal4 > brm* RNAi-2 background germaria cultured at 25 °C didn’t exhibited significant expansion of the UGCs, but when transferred to 29 °C the significant expansion of the UGCs phenotype was also exhibited (Figure S3A–D). Meanwhile, the knockdown of *bap170* or *bap180* with *c587-gal4* did not induce any obvious germline differentiation defects (Figure S3E). These results suggest that the Osa-containing BAP complex participates in germline differentiation control non-cell autonomously.

Furthermore, germaria with BAP complex component KD exhibited ectopic En in ECs (*brm* RNAi-1: 10/34 of total germaria, *snr1* RNAi: 6/45 of total germaira, *mor* RNAi-1: 18/32 of total germaria, *mor* RNAi-2: 5/41 of total germaria Figure 6F–J, white arrows; Figure 6L), leading to an *osa* KD phenocopy. The *brm* KD germaria also exhibited ectopic *dpp2.0-lacZ* in ECs (Figure S3F,G), suggesting that Brm may repress *dpp* transcription in ECs. Taken together, these results show that Osa regulates germ cell differentiation in a BAP-complex-dependent manner.

### 3.6. Osa Maintains Escort Cell Characteristics

Since EC protrusions are required to promote germ cell differentiation [8], we sought to determine if Osa regulates EC morphology. Thus, we introduced the *UAS-mCD8-GFP*/*+* into the background of *c587-gal4* to outline the morphology of ECs. In the control germaria, ECs extended protrusions to encapsulate the germ cells underneath (Figure 7A, white arrowhead). However, *osa* KD ECs failed to extend protrusions (Figure 7B,C white arrows). These results indicate that Osa may help to maintain EC protrusions. Moreover, we observed ectopic En staining and *dpp* transcription in *osa*-depleted ECs, which suggests a transition from EC to CpC. To determine whether *osa* mutant ECs completely switched their identities, we examined *c587-gal4* and *en* expression patterns in the control and *osa* RNAi ovaries. Normally, there is a mutually exclusive expression pattern of *c587-gal4* and *en* in control germaria (Figure 7A). Interestingly, *osa* KD induced En-positive *c587-gal4*-positive ECs (Figure 7B,C white arrows). Secondly, we checked the EC identity with that of another CpC marker, *hh-lacZ*. In the control germaria, *hh-lacZ* was expressed in TF and CpC at high levels and in ECs at low or undetectable levels. The average *hh-lacZ* intensity in ECs in the *c587*-gal4-driven *osa* depletion germaria was comparable to that in the control ones (Figure S4A–C). This result suggests that the expression level of *hh-lacZ* in the ECs was not altered upon *osa* depletion. Altogether, *osa-*depleted ECs displayed characteristics of both cell types in adult germaria. The *osa*-depleted EC obtained the ability of *dpp* production, which in turn activated BMP signaling activity outside the GSC niche. It should be noted that an enlargement of the GSC niche also induced UGC accumulation, as previously reported [64]. As *c587-gal4* was active in the precursor cells of both CpC and EC during the developmental stage, we could not rule out the possibility that *c587*-gal4-deriven *osa*-depletion induced an enlargement of the GSC niche. To rule out this possibility, we counted the CpC number in *osa*-depleted germaria and in the control ones. Interestingly, the CpC number was significantly reduced in the *osa*-depleted germaria, as highlighted by En staining (Figure 7D) and *hh-lacZ* (Figure S4D). Accordingly, the GSC number was also decreased by *c587-gal4-*driven *osa* RNAi (Figure S4E), supporting the decrease in CpC number. In summary, UGC accumulation in *c587-gal4-*driven *osa* RNAi germaria was elicited by the cell characteristics transition of ECs.

## 4. Discussion

Multiple studies have demonstrated that GSC progeny differentiation is a highly regulated process. Limiting *dpp* expression in the germline differentiation niche is crucial for proper germline development. In this study, we provided evidence that SWI/SNF component Osa depletion in escort cells (ECs) results in the blockage of GSC progeny differentiation. UGC accumulation occurs upon the loss of Osa in ECs containing a mixture of GSCs, pre-CB cells, and CB-like cells. Molecular and genetic studies suggest a link between ectopic *dpp* transcription in ECs and UGC accumulation elicited by *c587-gal4*-driven *osa* KD. Moreover, ectopic *en* expression in *osa*-depleted ECs partially contributes to upregulated *dpp* transcription. Osa in ECs also contributes to EC property regulation, such as regulation of EC protrusions. We also provided evidence that Osa in the anterior-most ECs may participate in GSC maintenance (Figure 7E).

In FACS-purified ECs, we observed significant upregulation in three of the four isoforms of *dpp* transcripts. This result was further confirmed by the *dpp* transcription reporter *dpp2.0-lacZ*. However, what motivates *dpp* transcription outside the GSC niche? Engrailed is known to be essential for *dpp* transcription in CpCs [62], and ectopic *en* expression in ECs leads to ectopic BMP signaling, resulting in the UGC accumulation phenotype [22]. These reports suggest that maintaining *en* expression in cap cells and suppressing *en* expression in ECs are critical steps for germline cell homeostasis. How *en* is regulated in cap cells and ECs remains a fundamental question in stem cell biology. Here, we identified that the Osa-containing BAP complex is required in ECs to limit *en* expression, and ectopic En in *osa*-depleted ECs also induces *dpp* transcription. Lsd1 was reported to limit *dpp* expression in ECs by repressing *en* ectopic expression in ECs [22], and there is a possibility that Osa cooperates with Lsd1 to regulate *en* expression in ECs. More investigation is needed to explore the molecular mechanism that determines how Osa regulates *en* expression in ECs.

A prior study reported that inhibiting the *brm* function in ECs alone did not produce any visible phenotype [57] and that *c587*-gal4-deriven depletion of *brm* can inhibit *dpp* derepression in ECs, which is caused by the *c587*-gal4-deriven depletion of PRC1 [26]. In this study, however, we obtained a contradictory result showing that *brm*-depletion in ECs induces UGC accumulation and that ectopic *dpp2.0-lacZ* activity outside the GSC niche uses the same *brm* RNAi line (*brm* RNAi-2) (Figure S3A–C,E,F). We previously studied the *c587*-gal4-driven *brm* RNAi line at 25 °C and failed to find obvious germline differentiation defects (Figure S3D). To increase KD efficiency, flies were initially kept at 25 °C until eclosion and then transferred to 29 °C for 3 days prior dissection, at which point the UGC accumulation phenotype could be detected. The phenotype was also confirmed with two individual *brm* RNAi lines (Figure 6A,B,K). This result is consistent with the finding that Osa regulates germline differentiation through the BAP complex, as the RNAi-mediated inactivation of either BAP component leads to UGC accumulation.

A recent study suggested that the GSC-contacting anterior ECs are essential for GSC maintenance [6]. We carried out an adult-specific KD of *osa* with *c587-gal4* to avoid the defects that arise during GSC niche formation. Further, *c587-gal4* was found to be restrictively expressed in ECs and early follicle cells in adult ovaries. Indeed, we observed significant UGC accumulation accompanied by a GSC loss phenotype. Thus, it is conceivable that Osa may be required in anterior ECs for GSC maintenance. Besides Dpp, some other factors, such as DE-cadherin and Wnt6, are also required in anterior ECs for GSC maintenance [6], but further investigation is needed to explore the molecular mechanisms by which Osa acts in adult ECs to modulate GSC maintenance. We also found that the CpC number was significantly reduced in *osa*-depleted germaria. It was reported that the activation of Notch signaling is essential for cap cell formation and adult cap cell maintenance [64,65]. During the late larva 3 (LL3) stage, newly formed TF secretes the ligand of Notch, Dl, thereby activating Notch signaling activity in the adjacent ICs and inducing cap cell formation. The overexpression of *Dl* in basal TFs increases the cap cell number [66], and *Dl* mutant basal TFCs induce fewer cap cells [65]. In the *Drosophila* adult midgut, Osa activated *Dl* expression in ISCs by binding to the *Dl* promoter region and then expanded Notch signaling activity in enteroblast, leading enteroblast to differentiate into enterocyte [37]. Osa might regulate the cap cell number by regulating the expression of *Dl* and other components of the Notch signaling pathway. However, further investigation is needed to explore this possibility.

## 5. Conclusions

In this work, SWI/SNF complex protein Osa was identified as a regulator of germline differentiation in the Drosophila female germline differentiation niche (posterior escort cells). Osa functions in escort cells to limit dpp transcription to prevent ectopic BMP signaling in the differentiating germline in part via suppressing EC-specific ectopic activation of Engrailed. Depletion of *brm* or other components of the BAP complex phenocopied the UGC accumulation phenotype, concluding a role that BAP chromatin remodeling complex in escort cells suppresses BMP signaling and facilitates cystoblast differentiation. In addition, Osa maintains the cellular extension of ECs which wraps and plays a role in promoting germline differentiation. Taken together, the epigenetic factor Osa plays an important role in controlling EC characteristics and germline lineage differentiation. We also provide evidence that Osa influences cap cell and GSC number, and Osa functions in adult ECs for GSC maintenance. The new findings would contribute to our understanding on how the *Drosophila* ovarian germline niche is established and in a broader view, the role of epigenetic machinery in defining the niche activity.

## Figures and Tables

**Table 1:** Primer sequences used for quantitative real-time polymerase chain reaction (qRT-PCR).

Gene	Sequence (5′-3′)
*dpp*	Forward primer: AGCCGATGAAGAAGCTCTACG
	Reverse primer: ATGTCGTAGACAAGCACCTGGTA
*dpp*-RA	Forward primer: TTGGAGCGTAACTGAGCGG
	Reverse primer: CGTTTGAAAAGTCGCCAGCA
*dpp*-RB	Forward primer: GTTTCGTACTTGGCTCATTGCG
	Reverse primer: CGTTTGAAAAGTCGCCAGCA
*dpp*-RC	Forward primer: GGGCGATCCATCCATCAAAC
	Reverse primer: CGTTTGAAAAGTCGCCAGCA
*dpp*-RE	Forward primer: TGCCAGATACGAAGAGTTGGG
	Reverse primer: CGTTTGAAAAGTCGCCAGCA
*rp49*	Forward primer: TCCTACCAGCTTCAAGATGAC
	Reverse primer: CACGTTGTGCACCAGGAACT

## Data Availability

All relevant data are within the paper and its Supporting Information files.

## Supplementary Materials

The following are available online at https://www.mdpi.com/2073-4425/12/3/363/s1. Figure S1: c587-gal4-driven osa RNAi knock down Osa in ECs efficiently. (A–C’) Germaria are stained for Osa (red) and GFP (green). mCD8-GFP is used to mark the EC cell membranes (detected by GFP). c587-gal4 is used for osa KD. The control germarium (A,A’) exhibit Osa staining in both germline and somatic cells, including ECs (indicated by white arrows). The osa KD (B–C’) germaria exhibited faint Osa staining in ECs (indicated by white arrows). (D) Graph shows the quantification of Osa staining mean fluorescence intensity ratio compared with the adjacent germline cells. Scale bar is shown in (A). Figure S2: Osa functions in both pupal stage and adulthood after EC formation for germline differentiation control. (A–H) Germaria are stained for α-spectrin (red), Armadillo (red) and DAPI (blue). c587ts is used for osa KD. (A, B) Flies are raised at 18 °C up to early pupal stage and then shifted to 29°C. The newly born females are dissected. (C,D) Flies are raised at 18 °C up to eclosion, and then shifted to 29°C for 14 days prior to dissection. (F–H) Flies are kept at 18 °C and dissected immediately after eclosion. The control germaria (A,C,F) exhibit normal number of CBs. The osa RNAi-1 germaria (B,D) exhibit UGCs accumulation. c587ts >osa RNAi-2 gives the similar phenotype and figures are not shown. (G,H) osa RNAi-1 (G) and osa RNAi-2 (H) groups exhibit normal procedure of germline differentiation. (E,I) Graph shows the quantification of UGCs per germarium. Error bars are presented as Mean ± SD. Several compressed z-sections are shown in (A–D,F–G). Scale bar is shown in (A). (J) Graph shows the quantification of GSC number per germarium. Figure S3: c587-gal4-driven Brm KD germarium exhibits UGCs accumulation and ectopic dpp2.0-lacZ activity in ECs. (A,B) Germaria are stained for α-spectrin (red), Armadillo (red) and DAPI (blue). CpCs are indicated by dashed circles. c587-gal4 is used for osa KD. The control germarium (A) exhibits normal number of CB. The brm KD germarium transferred to 29 °C (B) exhibits UGCs accumulation. (C,D) Graph shows the quantification of UGC per germarium. Error bars are presented as Mean ± SD. (E) Graph shows the quantification of UGC per germarium. Error bars are presented as Mean ± SD. (F,G) Germaria are stained for α-spectrin (red), Armadillo (red) and β-gal (green). dpp transcription is monitored by dpp2.0-lacZ (detected by β-gal). CpCs are indicated by dashed circles. The control germarium (F) exhibits specific dpp2.0-lacZ activity in CpCs. The brm RNAi-2 germarium (G) exhibits ectopic dpp2.0-lacZ activity in ECs. Several compressed z-sections are shown in (A,B,F,G). Scale bar is shown in (A). Figure S4: c587-gal4-driven osa RNAi didn’t change hh-lacZ expression level in ECs and result in reduced cap cell and GSC number. (A,B) Germaria are stained for α-spectrin (red) and β-gal (green). TF and CpCs are marked by hh-lacZ (detected by β-gal). CpCs are indicated by white dashed circles. c587-gal4 is used for osa KD. In control germarium (A) and osa KD germarium (B), hh-lacZ is highly expressed in TF and CpCs. C587>osa RNAi-2 gives the similar phenotype and figure is not shown. (C) Graph shows the quantification of hh-lacZ mean fluorescence intensity. (D) Graph shows the quantification of CpC per germarium. Error bars are presented as Mean ± SD. (E) Graph shows the quantification of GSC per germarium. Error bars are presented as Mean ± SD. (E) Graph shows the quantification of GSC per germarium.
