# Supplementary material for: The Osa-Containing SWI/SNF Chromatin-Remodeling Complex Is Required in the Germline Differentiation Niche for Germline Stem Cell Progeny Differentiation
Source: Genes (Basel). 2021 Mar 4;12(3):363. doi: 10.3390/genes12030363 (PMC7998989; doi:10.3390/genes12030363)
Supplement: Supplementary file 1 [file genes-12-00363-s001.pdf]

# Supplementary data

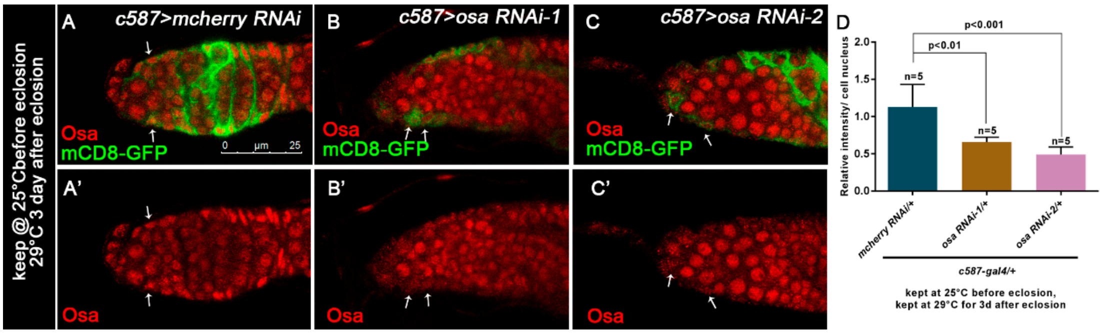


**Figure S1.** ***c587-gal4*-driven *osa* RNAi knock down Osa in ECs efficiently**

(A-C') Germaria are stained for Osa (red) and GFP (green). *mCD8-GFP* is used to mark the EC cell membranes (detected by GFP). *c587-gal4* is used for osa KD. The control germarium (A, A') exhibit Osa staining in both germline and somatic cells, including ECs (indicated by white arrows). The *osa* KD (B-C') germaria exhibited faint Osa staining in ECs (indicated by white arrows). (D) Graph shows the quantification of Osa staining mean fluorescence intensity ratio compared with the adjacent germline cells. Scale bar is shown in (A).

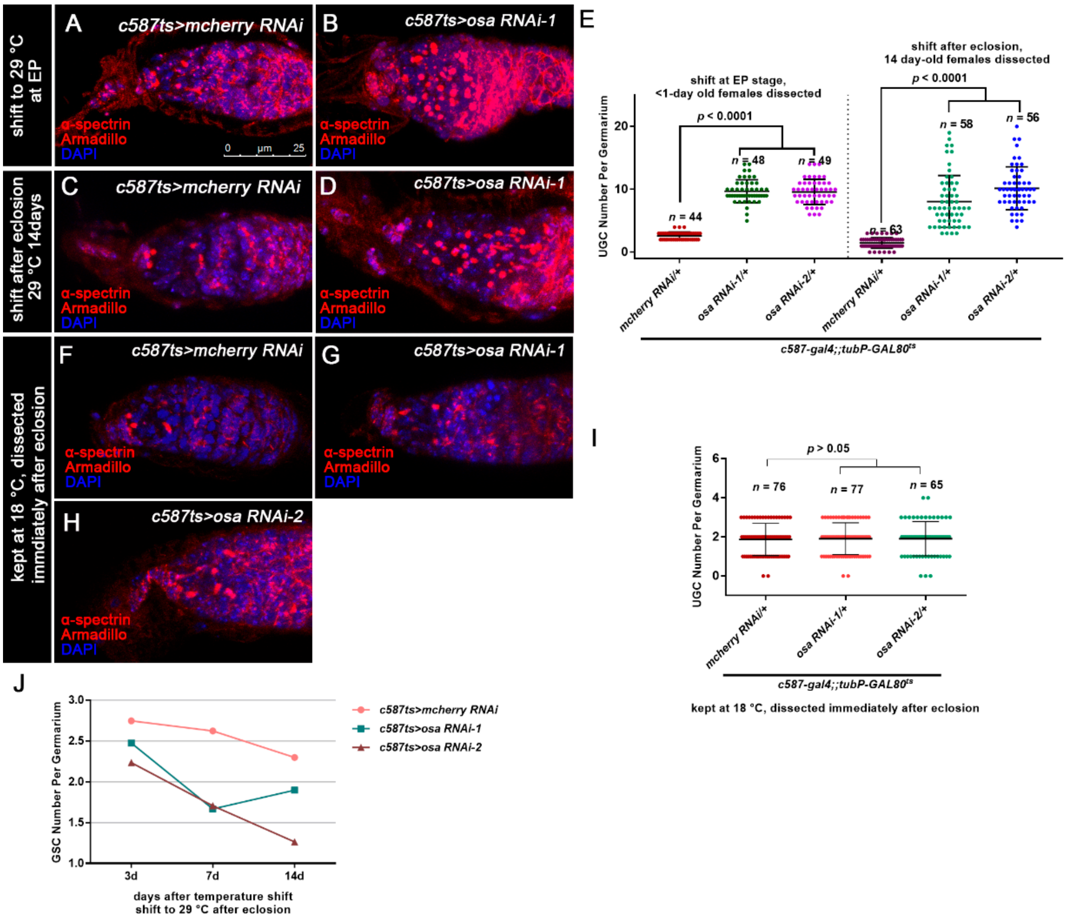


**Figure S2. Osa functions in both pupal stage and adulthood after EC formation for germline differentiation control.**

(A-H) Germaria are stained for α-spectrin (red), Armadillo (red) and DAPI (blue). *c587ts* is used for osa KD. (A, B) Flies are raised at 18°C up to early pupal stage and then shifted to 29°C. The newly born females are dissected. (C, D) Flies are raised at 18°C up to eclosion, and then shifted to 29°C for 14 days prior to dissection. (F-H) Flies are kept at 18°C and dissected immediately after eclosion. The control germaria (A, C, F) exhibit normal number of CBs. The osa RNAi-1 germaria (B, D) exhibit UGCs accumulation. *c587ts* >osa RNAi-2 gives the similar phenotype and figures are not shown. (G,H) *osa RNAi-1* (G) and *osa RNAi-2* (H) groups exhibit normal procedure of germline differentiation. (E,I) Graph shows the quantification of UGCs per germarium. Error bars are presented as Mean ± SD. Several compressed

z-sections are shown in (A-D,F-G). Scale bar is shown in (A). (J) Graph shows the quantification of GSC number per germarium.

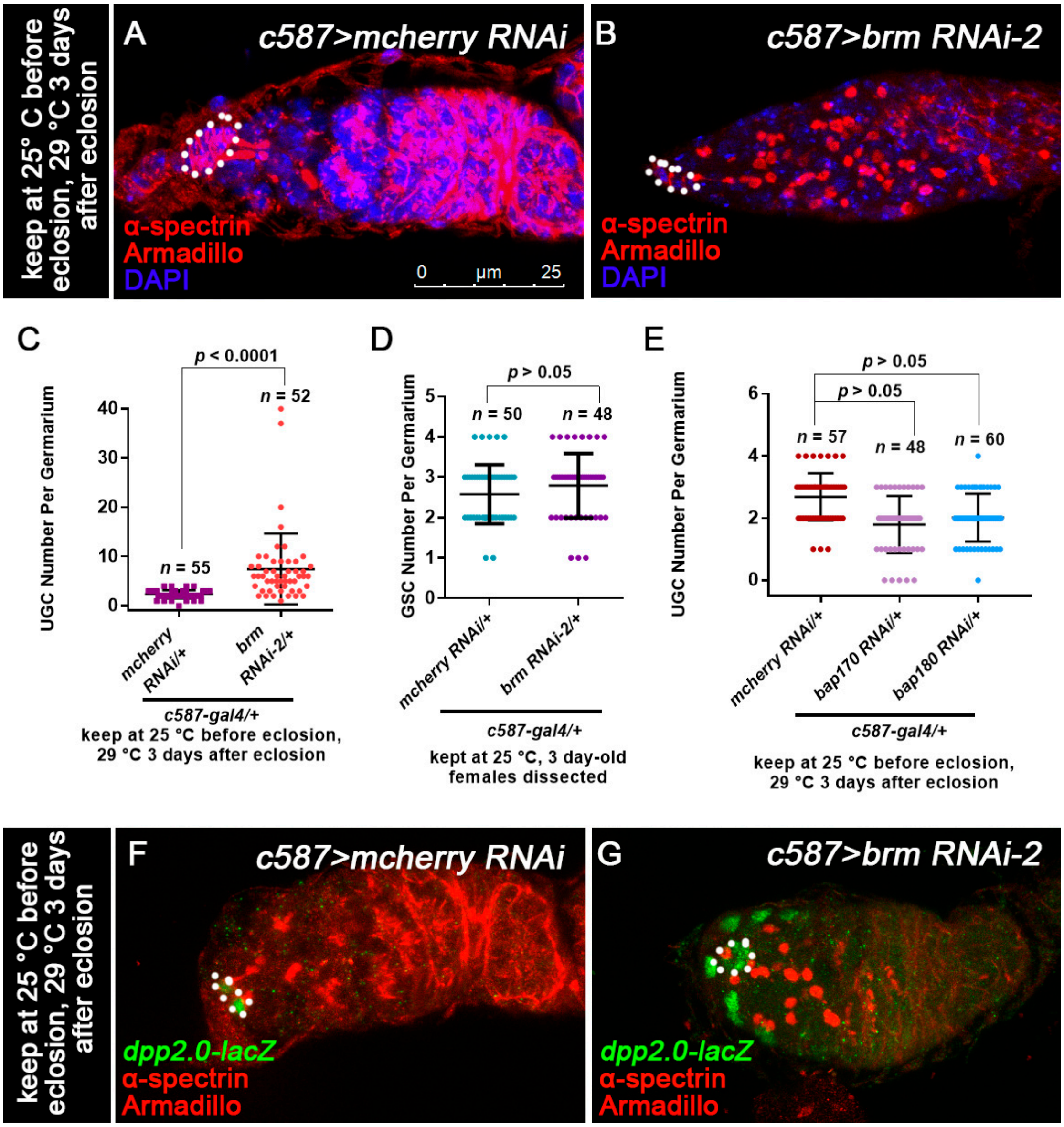


**Figure S3. *c587-gal4*-driven *Brm* KD germarium exhibits UGCs accumulation and ectopic dpp2.0-lacZ activity in ECs.**

(A, B) Germaria are stained for α-spectrin (red), Armadillo (red) and DAPI (blue). CpCs are indicated by dashed circles. *c587-gal4* is used for osa KD. The control germarium (A) exhibits normal number of CB. The *brm* KD germarium transferred to 29 °C (B) exhibits UGCs accumulation. (C,D) Graph shows the quantification of

UGC per germarium. Error bars are presented as Mean ± SD. (E) Graph shows the quantification of UGC per germarium. Error bars are presented as Mean ± SD. (F, G) Germaria are stained for α-spectrin (red), Armadillo (red) and β-gal (green). *dpp* transcription is monitored by *dpp2.0-lacZ* (detected by β-gal). CpCs are indicated by dashed circles. The control germarium (F) exhibits specific *dpp2.0-lacZ* activity in CpCs. The *brm RNAi-2* germarium (G) exhibits ectopic *dpp2.0-lacZ* activity in ECs. Several compressed *z*-sections are shown in (A, B,F, G). Scale bar is shown in (A).

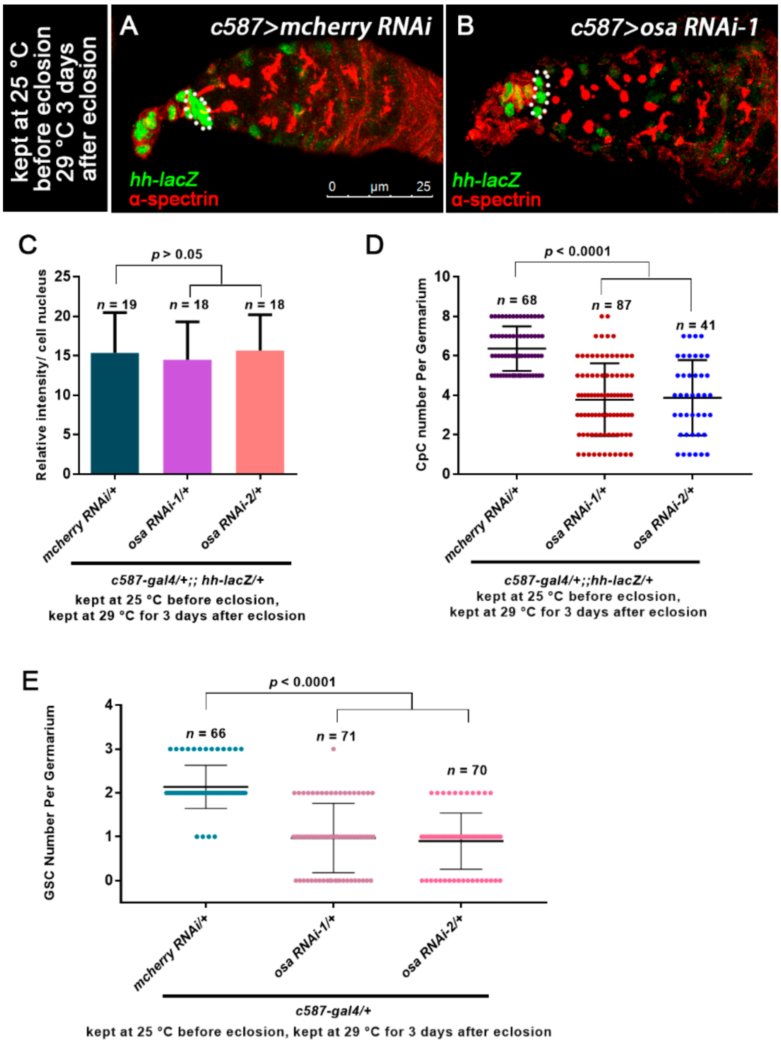


**Figure S4.** ***c587-gal4*-driven *osa* RNAi didn't change hh-lacZ expression level in ECs and result in reduced cap cell and GSC number.**

(A, B) Germaria are stained for α-spectrin (red) and β-gal (green). TF and CpCs are marked by *hh-lacZ* (detected by β-gal). CpCs are indicated by white dashed circles. *c587-gal4* is used for osa KD. In control germarium (A) and *osa* KD germarium (B), *hh-lacZ* is highly expressed in TF and CpCs. *C587>osa RNAi-2* gives the similar phenotype and figure is not shown. (C) Graph shows the quantification of hh-lacZ mean fluorescence intensity. (D) Graph shows the quantification of CpC per germarium. Error bars are presented as Mean ± SD. (E) Graph shows the quantification of GSC per germarium. Error bars are presented as Mean ± SD. (E) Graph shows the quantification of GSC per germarium..
